# Experimental evidence for adaptive divergence in response to a warmed habitat reveals roles for morphology, allometry and parasite resistance

**DOI:** 10.1002/ece3.10907

**Published:** 2024-02-07

**Authors:** Bethany A. Smith, Ana P. B. Costa, Bjarni K. Kristjánsson, Kevin J. Parsons

**Affiliations:** ^1^ School of Biodiversity, One Health & Veterinary Medicine University of Glasgow Glasgow UK; ^2^ Rosenstiel School of Marine, Atmospheric and Earth Science University of Miami Coral Gables Florida USA; ^3^ Department of Aquaculture and Fish Biology Hólar University Sauðárkrókur Iceland

**Keywords:** adaptive divergence, allometry, climate change, *Gasterosteus aculeatus*, geometric morphometrics, temperature

## Abstract

Ectotherms are expected to be particularly vulnerable to climate change–driven increases in temperature. Understanding how populations adapt to novel thermal environments will be key for informing mitigation plans. We took advantage of threespine stickleback (*Gasterosteus aculeatus*) populations inhabiting adjacent geothermal (warm) and ambient (cold) habitats to test for adaptive evolutionary divergence using a field reciprocal transplant experiment. We found evidence for adaptive morphological divergence, as growth (length change) in non‐native habitats related to head, posterior and total body shape. Higher growth in fish transplanted to a non‐native habitat was associated with morphological shape closer to native fish. The consequences of transplantation were asymmetric with cold sourced fish transplanted to the warm habitat suffering from lower survival rates and greater parasite prevalence than warm sourced fish transplanted to the cold habitat. We also found divergent shape allometries that related to growth. Our findings suggest that wild populations can adapt quickly to thermal conditions, but immediate transitions to warmer conditions may be particularly difficult.

## Graphical abstract

Geothermally warmed habitats provide a unique opportunity to test for routes of thermal adaptation in a natural warming experiment. We used a reciprocal transplant experiment to test for fitness consequences of morphological variation found in threespine sticklebacks (*Gasterosteus aculeatus*) originating from geothermal and cold habitats. We found evidence of unequal fitness consequences and some evidence that body shape and allometry play a role in this study system.

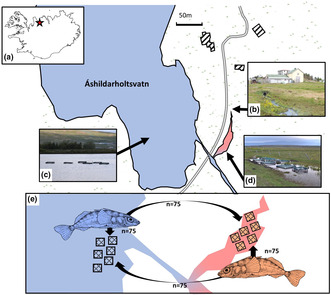

## 1 INTRODUCTION

The effects of climate change are expected to worsen within the next century, with a minimum of 1.5°C rise in the average global mean surface temperature (Lee et al., 2021). Increases in temperature will likely impose strong selection through effects on fundamental biological processes. Organisms tend to adapt to specific temperature ranges, with their ability to function dropping off at extremes (Pörtner & Farrell, 2008). As most fish are ectotherms, they are vulnerable to temperature changes, with increases posing a danger to their survival (Becker et al., 2018; Dudgeon et al., 2006; Pörtner & Knust, 2007). Even small increases in temperature that permit survival and reproduction could negatively affect the performance of individuals, as constraints and trade‐offs are placed on other traits. For example, temperature is an important factor for the growth of young fish (Friedland et al., 2005; Hurst et al., 2012; Teal et al., 2008; Todd et al., 2008) and the survival of juveniles (Beaugrand et al., 2003). This can trade‐off with their size and age at maturity (Attrill & Power, 2002; Cox & Hinch, 1997; Jonsson & Jonsson, 2004; Otero et al., 2012; Ottersen et al., 2006; Rogers et al., 2011; Schindler et al., 2005).

Temperature changes are also likely to affect host–parasite interactions to impact population fitness. For example, *Schistocephalus solidus* is a cestode macroparasite that infects stickleback as its second intermediate host (Hopkins & Smyth, 1951). Proportionate to body size, *S. solidus* infection can be massive with parasite mass reaching up to 92% of its host (Hopkins & Smyth, 1951). This comes at great cost to the host, including reduced growth, reproductive capability, impaired immune system function and increased risk of predation via behavioural manipulation (Barber et al., 2004; Franke et al., 2019; Grécias et al., 2018; Heins & Baker, 2003). The optimal temperature for *S. solidus* is higher than that of the host (Franke et al., 2017, 2019; Macnab & Barber, 2012), and the parasite can alter the thermal preferences of stickleback, so they seek out warmer waters (Macnab & Barber, 2012). Increased environmental temperatures may therefore benefit the parasite while negatively affecting the fitness of the host. Exactly how a mismatch in thermal optima between parasite and host could be compounded with the additional pressures from climate change is an open question.

Furthermore, increased temperatures not only impact fish directly but can also drive indirect effects by altering ecosystems. Indeed, warm and cold freshwater habitats differ in food web size, prey type and availability, structure and complexity (O'Gorman et al., 2012). Prey selection in fish is also impacted by temperature, potentially altering food web dynamics (O'Gorman et al., 2016). Such thermally induced changes in prey availability and optimal feeding strategy could interact to influence selective pressures under a novel thermal habitat, particularly on morphological traits that influence foraging success and swimming ability.

### 1.1 Impact of thermal conditions on phenotypes

While physiological and life history traits have been a focus for researchers interested in the adaptation of fish to increased temperature (Crozier & Hutchings, 2014), phenotypic effects are likely to be broad. For example, adaptive morphological variation has been intensively studied in fish in other contexts, where it relates to swimming performance, reproduction, mate selection, and foraging ability (Cooper et al., 2010; Head et al., 2013; Rowiński et al., 2015; Walker, 2010). Wild populations living in natural or man‐made warm habitats show morphological divergence when compared to non‐warmed temperature populations (Lema et al., 2019; Pilakouta et al., 2023; Rocamontes‐Morales et al., 2021; Rowiński et al., 2015). Growing evidence from lab experiments shows that fish morphology can be phenotypically plastic in response to temperature (Corral & Aguirre, 2019; Georga & Koumoundouros, 2010; Georgakopoulou et al., 2007; Marcil et al., 2006; Ramler et al., 2014; Sfakianakis et al., 2011), while heritable divergence in morphology related to temperature gradients has also been found (Marcil et al., 2006; Pilakouta et al., 2023). Most commonly seen in both lab and field examples is a deepening of body shape with increased temperature (Georgakopoulou et al., 2007; Lema et al., 2019; Marcil et al., 2006; Pilakouta et al., 2023; Rowiński et al., 2015). This consistency in morphological divergence suggests that such changes could be adaptive (Pilakouta et al., 2023), and/or influenced by biased developmental responses (Parsons et al., 2020). Temperature‐related morphological variation is relatively new, and tests of its fitness consequences, such as those performed by Ackerly and Ward (2016), have been rare.

Furthermore, understanding the relationship between morphological variation and thermal habitat could benefit from a developmental perspective (Campbell et al., 2017, 2021). Indeed, temperature commonly affects growth rates in fish (Brett, 1979), suggesting that scaling relationships (allometry), which arise from differing relative growth rates between body parts, or with size, could be impacted (Casasa & Moczek, 2019; Savageau, 1979). Allometry itself is known to evolve in response to selection (Houle et al., 2019; Pélabon et al., 2014), suggesting it could be influenced by temperature variation. Temperature‐induced body shape variation in fish can be dependent on size (Lema et al., 2019; Rowiński et al., 2015). However, whether such allometry adaptively diverges in response to thermal conditions is unclear.

### 1.2 Wild systems for studying thermal effects on phenotypic variation

Slow or inadequate adaptation to even low‐level warming could result in an increased vulnerability to a range of threats (Becker et al., 2018), and possibly local extinction. While populations may simply leave unfavourable conditions, some face particular risks, such as in freshwater fish where there can be relatively few opportunities to migrate away (Dudgeon et al., 2006). Therefore, it will be important to test how fish populations in nature adapt, where both direct and indirect effects of warming exist. While rare, some systems can enable examination within predicted future conditions. For example, geothermal habitats offer opportunities for studying temperature effects on a range of extant organisms (O'Gorman et al., 2014; Pilakouta et al., 2020). The wide‐ranging thermal gradients that can occur over short physical distances within these habitats allow for comparisons of ‘warm’ and ‘cold’ populations without the same degree of confounding factors (e.g. photoperiod, geology, overall ecosystem makeup and large amounts of genetic drift between populations) found over large latitudinal or altitudinal gradients. Such study systems are a highly valuable complement to laboratory‐based experiments.

Geothermal habitats are common in Iceland, resulting in extreme temperature gradients across just a few meters, often with no physical barriers (Jónasson et al., 1977; O'Gorman et al., 2012, 2014). Prior research has identified several of these habitats populated by the threespine stickleback (*Gasterosteus aculeatus*) (Pilakouta et al., 2020) – a well‐known model species for ecology and evolution (Hendry et al., 2013). In these locations cold and geothermal (warm) habitats can differ by more than 10°C at a given time (Millet et al., 2013; Pilakouta et al., 2020). Ongoing research has found that these warm and cold sourced stickleback populations differ in diet, sociability, morphology and metabolic rate (Pilakouta et al., 2020, 2022, 2023; I. Fisk, G. Lawson‐Duck and K. J. Parsons, Unpublished data). Heritable morphological divergence between these stickleback takes the form of a deeper body, more subterminal mouth, steeper craniofacial profile and a longer second dorsal spine in warm sourced fish (Pilakouta et al., 2023). Furthermore, the repeated nature of this divergence across independent populations suggests that adaptative divergence has occurred, but no direct experimental tests of adaptation have been performed.

Here, using stickleback populations inhabiting warm and cold thermal habitats, we tested for evidence of adaptive divergence. Specifically, using a field‐based reciprocal transplant experiment (Blanquart et al., 2013; De Villemereuil et al., 2016) we predicted (1) that indicators of fitness, would be improved, in the form of higher growth and survival, and lower parasite prevalence, when fish were within their native habitat relative to a non‐native habitat. Parasite prevalence may also have a more complex interaction, due to the likelihood that the conditions of the warm habitat will benefit parasite fitness over that of the host fish, and the potential for warm sourced fish to have adapted to this pressure. We also predicted (2) that morphological variation would relate to growth and (3) that increased growth in the non‐native habitat would be associated with body shapes more similar to that of natively sourced fish. Finally, we predicted (4) that morphological allometry could have an impact on growth.

## 2 METHODS

### 2.1 Ethics statement

All activities adhered to the ASAB/ABS Guidelines for the Use of Animals in Research, the institutional guidelines at the University of Glasgow, and the legal requirements of the UK Home Office (Project Licence P89482164). In Iceland, permissions were obtained from local landowners for sampling and experimental setup. All animal storing and handling was conducted under the animal care licence (FE‐1051) of the Hólar University Research Station, Verið, issued by the Icelandic food and veterinary authority.

### 2.2 Study system

**FIGURE 1:**
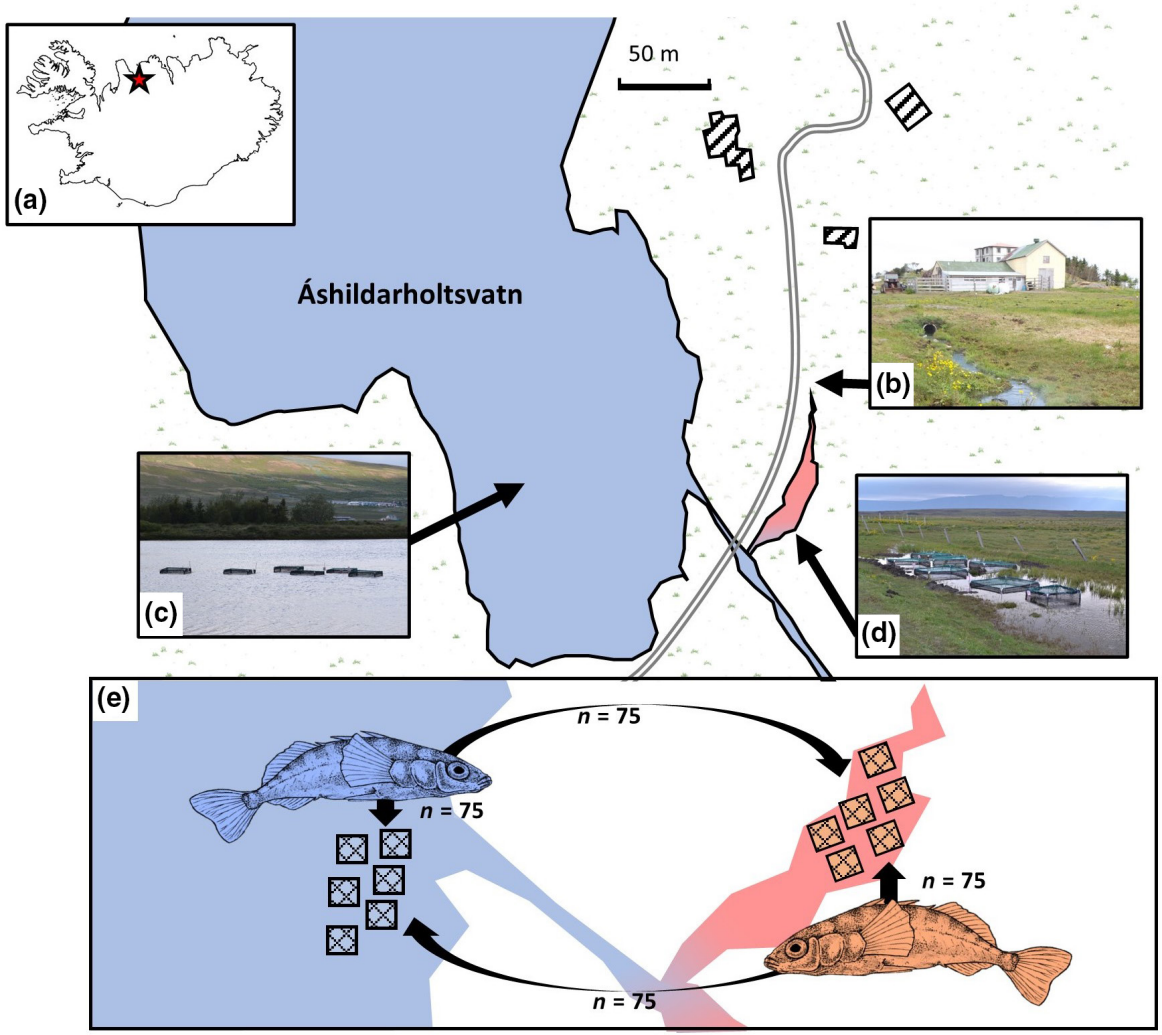
Geothermal‐ambient study system used in this experiment (a) location of the Áshildarholtsvatn habitat pair in the North of Iceland (red star), (b) photograph showing the warm water outlet pouring into the warm habitat, (c) cold habitat with cages, (d) warm habitat with cages, (e) experimental design of the reciprocal transplant experiment, showing transplantation of warm (pink) and cold (blue) sticklebacks to their native and opposing habitats.

We tested for adaptive thermal habitat divergence by performing a reciprocal transplant experiment at Áshildarholtsvatn (65°43′30.3″ N, 19°36′02.5″ W, Figure 1a) – a lake located near Sauðárkrókur in Northern Iceland. Adjoining Áshildarholtsvatn (ASHN) is a small pond fed by hot‐water runoff (source temperature ~ 45.5°C) from nearby residential geothermal heating, houses built in the 1940s, installed within the past 70 years (Figure 1b). This pond eventually flows into a stream that runs off from the lake, creating an abrupt thermal gradient between the warm and cold habitats (Figure 1c,d). The pond created by the hot water runoff (hereafter called the warm habitat) experiences temperatures around 10°C higher than the cold‐water stream and lake (hereafter called the cold habitat) year‐round (Pilakouta et al., 2023). At the start of the experiment in June 2019 the temperatures were an average of 15.8°C for the warm habitat and 8.7°C for the cold habitat.

The cold habitat is larger and deeper than the warm habitat and differs in water chemistry in the concentrations of phosphate, magnesium, iron, silica and calcium, but is otherwise similar in measured parameters (see Table S1). Despite the relatively young age of this system, and the lack of a physical barrier between habitats, evidence of heritable divergence has been found (Pilakouta et al., 2023).

### 2.3 Fish collection and processing

A total of 430 fish from each habitat were caught over 3 days using unbaited minnow traps, laid for 24 h. The warm and cold source fish were housed separately in four 20 L buckets at Verið, Hólar University Research Station, in a flow‐through system under a temperature of 12.5°C (±1°C) which was intermediate to field site conditions. Fish were fed with 0.4 mm aquaculture feed every other day. Fish with noticeable swelling due to *Schistocephalus solidus* infection were excluded, although it was not possible to accurately determine infection status prior to the experiment. To increase the scope for growth during the experiment we chose juvenile sticklebacks of a specific size range and lacking signs of sexual maturity (gravidity or male colours). Populations of wild stickleback vary considerably in standard length at sexual maturity, with reported size ranges typically contained between 32 and 90 mm (Baker et al., 2015; DeFaveri & Merilä, 2013; Millet et al., 2013; Narver, 1969; Walker, 1997). Mature stickleback captured from another Icelandic lake have been found to average 53.6 ± 1.01 mm from the warm habitat, with stickleback from their corresponding cold habitat averaging 55.0 ± 1.01 mm (Millet et al., 2013). From lab‐reared F1 Áshildarholtsvatn stickleback we observed average lengths of 53.9 mm (SD 6.74) and 58.8 mm (SD 4.75) in sexually mature cold and warm sourced stickleback respectively. Therefore, we chose a mean starting length of 40.6 mm (SD 3.4), which corresponded with an average weight of 0.75 g (SD 0.22).

Within 3 days of capture the experimental fish were anaesthetised using phenoxyethanol, weighed and photographed (for morphometric analysis and standard length measures) using a Nikon D3100 camera (Nikon, Tokyo, Japan) and then tagged with two visual implant elastomer tags (Northwest Marine Technology, Inc., Anacortes, USA) for re‐identification following the conclusion of the reciprocal transplant experiment. The tagged fish were allowed to recover in captivity (minimum 10 days, maximum 13 days), with any dead fish being removed, identified and replaced with a newly tagged fish if necessary (36 fish were replaced in this manner). Fish were not re‐weighed after their time in the lab in order to reduce handling time and stress as much as possible.

### 2.4 Cage set up

Reciprocal transplant cages consisted of black 5 mm Fryma Mesh (Collins Nets LTD, Dorset, UK) stretched over a cuboid skeleton of 32 mm PVC pipes. The 5 mm hole size of the Fryma Mesh was selected to allow for small fish to be used in the experiment, while also allowing invertebrates to pass through, as in previous stickleback transplant experiments (Hatfield & Schluter, 2006; Kaufmann et al., 2017; Stutz et al., 2015). The six cages intended for the cold habitat were 1 × 1 × 1 m, while the six warm habitat cages were of approximately the same volume but were 1.42 × 1.42 × 0.5 m in dimensions. These differences in dimensions were due to the shallow nature of the warm habitat. Warm habitat cages were placed close to the inlet of warm water where sticklebacks were naturally found and spaced approximately 50 cm apart to allow for water flow. Cold habitat cages were placed ~1 m apart at the shore of the lake at a depth just short of 1 m. All cages were seeded with sediment from their habitats and one cluster of native plants to provide shelter. All cages were then left for 4 days after placement to allow sediment to settle until the water was clear.

### 2.5 Reciprocal transplant

Warm and cold sourced fish were alternated across a set of cages in both habitats to evenly distribute treatment types across possible environmental gradients. Three replicate cages were used for each of the four treatment types (cold source fish transplanted to the warm habitat, warm to cold, warm to warm, and cold to cold). Tagged fish were selected haphazardly from those housed in the lab, but tag codes for each individual in a cage were recorded to enable re‐identification. Each cage housed 25 fish, resulting in 75 fish per treatment type and a total of 300 fish used in the experiment (Figure 1e). Selected fish were released into the cages which were then covered with Nylon anti‐bird netting with 22.5 mm^2^ holes. The transplant experiment ran for 30 days from mid‐June to mid‐July of 2019, with a take‐down period of 5 days. During the experiment, all cages were checked three to four times a week for structural integrity and the temperature measured at three points along the shore of each habitat. At the end of the experiment stickleback were collected from cages with the use of unbaited minnow traps checked hourly. The cages were then removed from the habitat, and the sediment within the cages was checked for the presence of remaining fish. All cages were found to be intact at the end of the experiment; thus, unrecovered fish were assumed to have died. Recovered fish were transported back to the laboratory, euthanised with an overdose of phenoxyethanol, re‐identified by elastomer tags, re‐photographed for length measurements and weighed. Fish were then dissected for assessment of *Schistocephalus solidus* parasite status (infected/uninfected). As this was only a preliminary investigation, no further parasite data was recorded. Sex was not determined as many individuals were immature and could not be confidently identified as male or female.

### 2.6 Gathering morphological data

**FIGURE 2:**
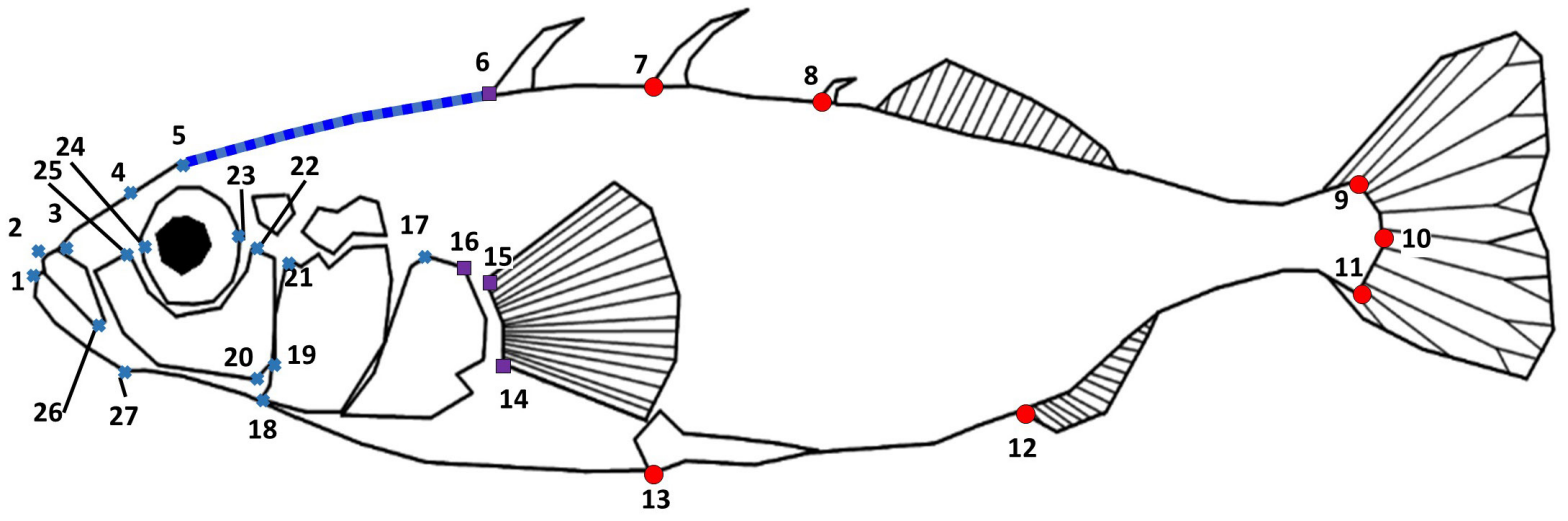
The 27 landmarks and semilandmark curve used in analysis of stickleback shape. 1 – anterior tip of the mandible, 2 – anterior tip of premaxilla, 3 – maxilla, 4 – nares, 5 – frontal, directly above eye, 6, 7 & 8 – anterior bases of first, second and third dorsal spines, 9, 10 & 11 – caudal peduncle, 12 – anterior base of anal spine, 13 – base of pelvic spine, 14 & 15– insertion points of pectoral fin, 16 & 17 – dorsal and anterior corners of pectoral girdle, 18 – junction of head to body on ventral midline, 19 & 21 – ventral and dorsal anterior corners of operculum, 20 & 22 – ventral and dorsal corners of pre‐operculum, 23 & 24 – posterior and anterior edge of eye, 25 – ventral corner of lacrimal, 26 – Posterior end of premaxilla, 27 – posterior end of angular. Fifteen sliding landmarks were placed between landmarks 5 and 6 (blue line) for forehead morphology. Blue crosses are head landmarks, red circles are body landmarks, and purple squares were used in both head and body data set.

To assess morphological variation, landmarks were collected for each fish from photographs taken prior to the start of the experiment using TPSdig2 version 2.31 (Rohlf, 2008). To minimise potential biases in landmarking, photographs were randomly arranged using the *randomly order specimens* function in TPSUtil 1.78 (Rohlf, 2015). A total of 27 landmarks (LMs) and a curve of 15 sliding semi‐landmarks were placed on each fish (Figure 2) with an eye to capture shape variation found to be important in warm‐cold stickleback divergence (Pilakouta et al., 2023). In a small number of photos (*n* = 11) the mouth of the fish was slightly open affecting one landmark (landmark 1, Figure 2). Also, one cold to warm transplant fish was found to be missing the first dorsal spine (landmark 6). Therefore, in these cases the affected landmark was designated as missing and its position was estimated using the thin‐plate spline method to estimate missing landmarks following Adams et al. (2020) using the *missing.landmark* function in the R package *Geomorph* version 3.1.3 (Adams et al., 2020; Adams & Otárola‐Castillo, 2013). A small number of fish had more than three dorsal spines, in which case only the first three spines were landmarked. Body length was measured as standard length using the distance between landmarks 2 and 10 (Figure 2).

### 2.7 Data analysis

#### 2.7.1 Testing for evidence of adaptive divergence

All statistical analyses were conducted within the R 4.2.2 statistical language (R Core Team, 2022). We report findings with an alpha value between 0.1 and 0.05 as suggestive, but 0.05 was used as an indicator of statistical significance for all analyses. To test whether fitness proxies would improve when fish were within their native habitat (prediction 1), we used survival, parasite infection, and growth as response variables. Survival and parasite presence were measured as binary variables, and chi‐squared tests were used to test for differences between treatment groups. Further analysis using binomial family GLMs tested for the effects of transplant treatment on each survival and parasite infection. A simple candidate model was created for each analysis, with the variables of source habitat, destination habitat and the interaction between the two, as shown below.

survival~source habitat*destination habitat

parasite infection status~source habitat*destination habitat

A further 14 candidate models were created for each analysis, based on the simple model, each including and excluding additional variables to control for the effects of starting size (starting weight and length), cage effects (cage nested within destination) and potential interactions between each (Table S2). Model selection was performed in order to assess whether cage effects and starting size were significant for inclusion in the final model. Model selection was based on AIC values obtained from the ANOVA function in R (package *stats* version 4.2.2 (R Core Team, 2022)). Models with the lowest AIC value were taken to represent the best fit to the data, but when two models had AIC value within two of each other, we selected the simpler model following Burnham and Anderson (2002). Model permutations and selected models are shown in Table S2.

Growth, measured as changes in weight and length, was also examined in relation to the impacts of source, destination and their interaction using GLMs. However, to account for the possibility that fish of different starting sizes would exhibit varying growth rates, we first standardised changes in weight and length against their respective size at the beginning of the experiment using a linear regression to obtain residuals that were then used for GLMs. Linearity of growth was checked by examining fitted versus residual plots and was found to be linear; therefore, no log transformation was applied. A simple candidate model was created for each analysis, with the variables of source habitat, destination habitat and the interaction between the two, as shown below.

residual weight change~source habitat*destination habitat

residual length change~source habitat*destination habitat

A further five permutations of each of these models including and excluding additional variables to control for cage effects (cage nested within destination) and parasite effects (*S. solidus* infection status) were created as a basis for model selection, which was performed in the same manner as for the binomial models (Table S2).

Notably, weight and length change data were not combined into a single condition factor as this may reduce the ability to explain changes in size and cause difficulties comparing populations with potentially different weight‐length relationships (Froese, 2006). Additionally, because fish did not have *S. solidus* parasites removed before weighing, and due to the potentially substantial sizes of these parasites, weight values at the end of the experiment (unlike standard length) may not have been wholly attributable to the weight of the fish alone. For both the binomial and GLM models, findings of interactions between source and destination effects would be indicative of adaptive fitness differences between warm and cold sourced fish, supporting our prediction of adaptive divergence.

#### 2.7.2 Testing relationships between growth and shape

As anatomical regions can have both differing functions and developmental origins (Parsons et al., 2011), and because body shape was potentially susceptible to influences from parasite prevalence, shape analysis was performed on three sets of landmark data: the head, the posterior body, and both combined (Figure 2, see also Wund et al., 2008). The landmarks used for the head subset were 1–6, 14–27, and the sliding landmark curve, while the posterior body subset consisted of landmarks 7–16, with both subsets comprising the whole fish, i.e., total body shape (Figure 2). Each set of landmarks was standardised for variation in size, translated and rotated to minimise interindividual landmark distances using a generalised Procrustes analysis implemented from the *gpagen* function in *Geomorph* with sliding semilandmark positions optimised to minimise bending energy.

To address prediction 2 and determine the relationship between morphology, our experimental factors and growth, each landmark set was modelled using a Procrustes ANOVA in *Geomorph*. Here, landmarks were used exclusively from fish that survived the experiment (*n* = 269). For each Procrustes ANOVA source, destination, log centroid size (to account for static allometry) and one growth measure for each model (either residual weight or length change) were tested for effects on morphological shape using the *ProcD.lm* function with type III sums of squares (see base model design below).

$$\text{Shape}\sim \text{residual weight change}*\text{source}*\text{destination}*\log\left(\text{centroid size}\right)$$

$$\text{Shape}\sim \text{residual length change}*\text{source}*\text{destination}*\log\left(\text{centroid size}\right)$$

To allow for model selection and to assess whether there was a need to control for cage effect, a second version of each model was also made with the additional variable of cage (nested within destination). Model selection was performed using a nested ANOVA through the ANOVA function (package *stats* version 4.2.2) whereby if cage effects did not significantly change results the simpler model was chosen.

To address prediction 3, that increased growth in the non‐native habitat would correspond to morphological shape resembling the average native shape, we visualised both (1) the average divergence in shape between warm and cold source stickleback, and (2) the relationships between shape and growth measures (weight change or length change) using deformation grids. Deformation grids were created using the *PlotReftoTarget* function in *Geomorph*, which generates thin‐plate spline deformation grids plotting the difference in shape of a target specimen in relation to a reference specimen. Thus, a deformation grid representing how warm sourced fish differed from a cold sourced fish was created by using the mean shape of cold sourced fish as a reference against the mean shape of warm sourced fish as the target. Also, differences in shape between the best and worst performing fish (measured as length change or weight change) in each treatment group were estimated from growth and deformation grids created from growth values found to relate to shape in our Procrustes ANOVAs. We used the *Geomorph* function *shape.predictor* to estimate shape against minimum and maximum growth values. Deformation grids were created where one extreme of the scale would represent the shape of fish with the “best” growth (e.g. greatest increase in length), while the other end of the scale represented the shape of fish with the “worst” growth (e.g. lowest increase in length). To accentuate differences, shape deformation was magnified by 3×.

#### 2.7.3 Testing relationships between growth and allometry

To address prediction 4, that adaptive morphological variation could be attributed to allometry, we assessed the contribution of allometry to shape divergence by testing whether size/shape relationships were different between warm and cold source fish. This was done as a follow‐up to our Procrustes ANOVAs to further understand whether potential interactions between size and shape were due to differential allometry between fish from different thermal sources. Therefore, to address whether size simply differed between fish from different thermal sources we first tested whether geometric centroid size and starting length differed between warm and cold populations using a *t*‐test (*t.test* function, package *stats* version 4.2.2). We also tested for differences in the variation of centroid size and starting length between warm and cold populations using a Levene's test using the *leveneTest* function from the *car* package in R (Fox & Weisberg, 2019; Levene, 1960). Following this, a test for allometric differences between fish from the two thermal habitats was performed for each set of landmarks using ANOVA to compare a unique allometric *ProcD.lm* model (with an interaction between log centroid size and source) to a null common allometric *ProcD.lm* model (with no interaction between log centroid size and source). Due to the potential for *S. solidus* to alter the centroid size of fish, a similar model was created but used the starting length of fish as a measure of size. We also visualised allometric relationships using *Geomorph*'s *plotAllometry* function to create two plots for each analysis. The first plot, using the option ‘*PredLine*’, plots the first principal component of predicted values versus size from the calculated fitted values from the selected *procD.lm* fit in the test of allometric relationships (unique vs. common). The second plot, using the option ‘*RegScore*’, plots standardised shape scores, calculated from the regression of shape on size, against size.

Finally, to test for evidence of adaptive allometric variation we compared shape between survivors and fatalities. Specifically, rather than using all fish (*n* = 300) this involved separately testing for differences in morphological shape between survivors and fatalities in the treatment experiencing the lowest survival rate (one treatment group, total sample size *n* = 75) using a Procrustes ANOVA with survival and size (centroid size and length) as explanatory variables. Finally, to rule out the possibility that survival was determined by size, a standard *t*‐test of centroid size comparing fish that survived or died was performed using the *t.test* function. We also tested for a difference in the variation of centroid size and starting length between stickleback that survived or died during the experiment using a Levene's test from the *leveneTest* function within the *car* package in R (Fox & Weisberg, 2019; Levene, 1960). We visualised allometric shape variation that differed between survivors and fish that died in this group using deformation grids generated by predicting shape against minimum and maximum log centroid size and plotted with *Geomorph*'s *PlotReftoTarget* using the same method as used for the previous deformation grids. These deformation grids were magnified by 3× to enable clear visualisation of allometric divergence. Plots of allometric relationships were created using p*lotAllometry* as before.

## 3 RESULTS

### 3.1 Testing for evidence of adaptive divergence

**FIGURE 3:**
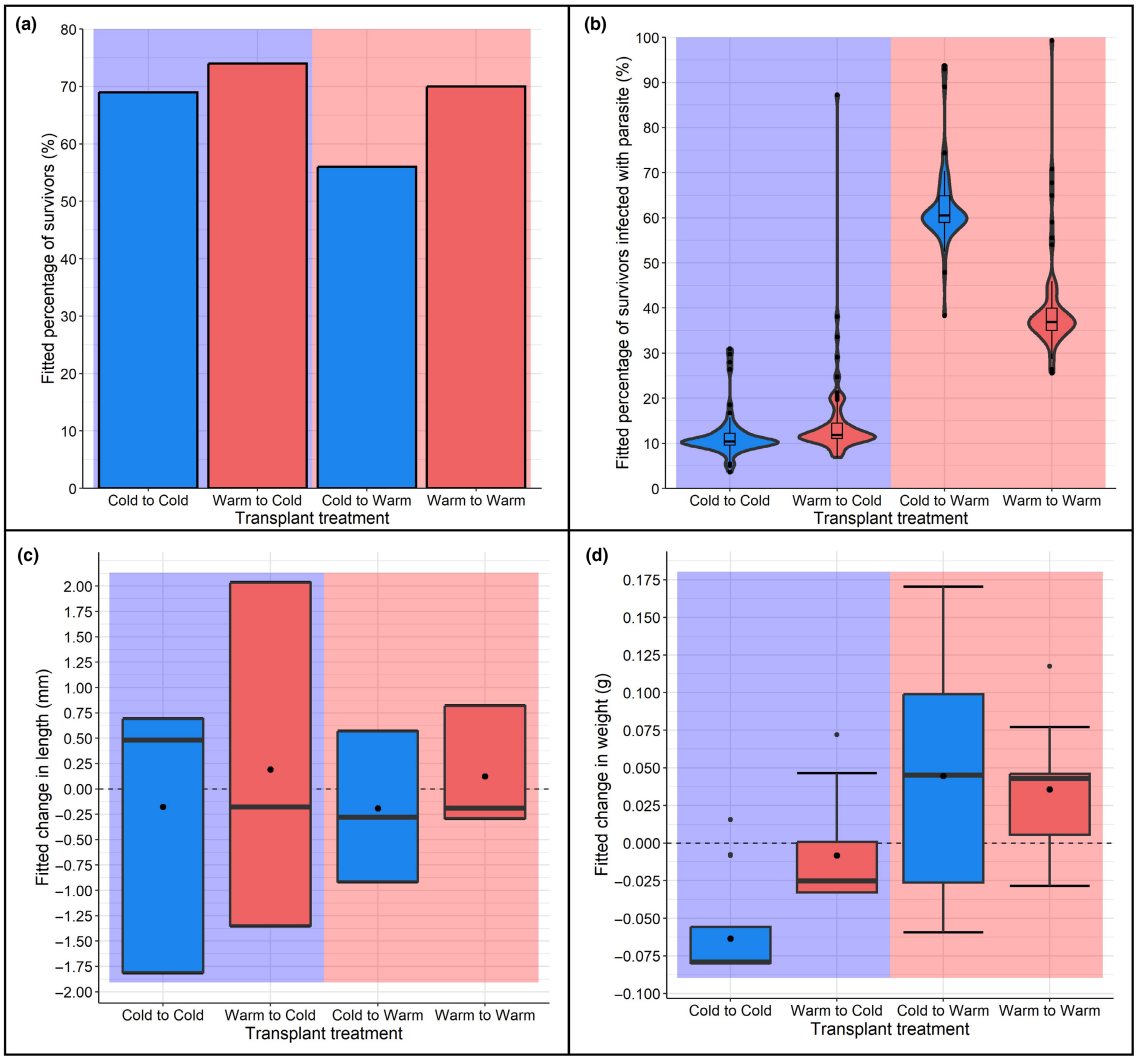
Fitted values of performance measures from model analysis for (a) survival (mean percentage of fish recovered per cage per treatment) and (b) *Schistocephalus solidus* prevalence (mean percentage of recovered fish found to be infected per cage per treatment). Treatment designated by the thermal source (warm and cold) of the fish and thermal habitat destination (warm and cold). As the simplest model design was selected for the survival analysis (survival ~ source*destination), no variation in fitted values was present between individuals within each treatment type. Box and whisker plots (c) and (d) display fitted variation in (c) residual length changes (mm) and (d) residual weight changes (g), that occurred with experimental treatments in the reciprocal transplant experiment involving natural cold and warm habitats. Top and bottom hinges represent 25th and 75th percentile, centreline represents 50th. Black dot displays mean residual weight change. Whiskers give 95% confidence interval. Blue and red filled boxes represent the cold and warm sourced fish respectively, blue and red backgrounds represent the cold and warm destination habitats, respectively.

Overall, survival was high (89.7%), but survival rates differed among transplant treatment types ($X_{\left(1,N=300\right)}^{2}$ = 26.298, *p* < .05). Cold sourced stickleback transplanted to the warm habitat experienced the lowest survival rate at 74.7% as compared to ≥92% survival rates in other groups (warm to warm: 93.3%, cold to cold: 92% and warm to cold: 98.7%; Figure 3a). The best supported survival binomial model showed that destination and source had an effect on survival (Table 1).

**TABLE 1:** Results of binomial family GLMs (model permutation selected by AIC) testing for the effect of a reciprocal transplant experiment on survival and parasite infection of threespine stickleback in Iceland.

Binary fitness proxy measure and variables	Est. coefficients	SE	*Z*	*p*
Survival				
Source	1.862	1.093	1.703	**.089**
Destination	−1.361	0.502	−2.714	.007**
Source × Destination	−0.304	1.216	−0.250	.803
(Intercept)	2.442	0.426	5.738	<001***
Parasite infection				
Source	0.111	0.514	0.217	.828
Destination	2.611	0.478	5.461	<.001***
Source × Destination	−1.080	0.636	−1.698	**.090**
Start weight	−19.257	9.355	−2.058	.040*
Start length	−0.280	0.170	−1.644	.100
Start weight × start length	0.495	0.230	2.152	.031*
(Intercept)	8.691	6.924	1.255	.209

*Note*: *p* values below .1 are highlighted in bold. *p* values <.05 and ≥.01 are indicated with a single asterisk, <.01 and ≥.001 with two asterisks and <.001 with three asterisks. Rows highlighted in blue represent interaction combinations that address the question of whether the fitness proxy measure in question relates to transplant treatment.

The prevalence of *S. solidus* differed between fish source and destination ($X_{\left(1,N=269\right)}^{2}$ = 50.217, *p* < .05). Fish caged in the warm habitat were more likely to be infected than fish caged in the cold habitat with 40% of warm and 62.5% of cold source surviving stickleback infected, while in the cold habitat only 11.6% of cold and 14.9% of warm source surviving stickleback were infected (Figure 3b). The selected binomial parasite prevalence model indicated effects from source, destination and the interaction between source and destination, suggesting adaptive divergence in relation to parasite resistance (Table 1).

The transplant experiment also impacted fitness proxies, as measured through residual weight change (Table 2 and Figure 3c,d). Model selection favoured a GLM with source, destination, cage (nested within destination) and parasite infection status as factors, with adaptive divergence indicated through an interaction between source and destination (Table 2). Fish transplanted to the warm habitat were more likely to gain weight (mean residual weight change = 0.04 g), while in the cold destination weight loss was more likely (mean residual weight changes = −0.06 g) (Figure 3d). However, this was not equal across sources with fitted weight change indicating that warm sourced fish transplanted to the cold habitat were more likely to lose weight than warm sourced fish in the native habitat (Figure S1).

**TABLE 2:** Results of GLMs (permutation selected by AIC) testing the effect of the reciprocal transplant experiment of threespine stickleback in Iceland on growth measures (residual length and weight change).

Fitness proxy measure and variables		Est. coefficients	SE	*T* value	*p*
Residual weight change					
Source		0.054	0.027	1.975	.049*
Destination		0.178	0.031	5.710	<.001***
Source × Destination		−0.181	0.041	−4.439	<.001***
Parasite infection status		0.072	0.014	5.100	<.001***
Cage (Nested within destination)	# 2	−0.158	0.031	−5.024	<.001***
	# 3	0.034	0.028	1.217	.225
	# 4	−0.120	0.032	−3.728	<.001***
	# 5	0.075	0.028	2.678	.008**
	# 10	0.026	0.027	0.958	.339
	# 11	−0.001	0.028	−0.020	.984
	# 12	−0.008	0.027	−0.284	.7769
	# 13	0.023	0.028	0.825	.4102
(Intercept)		−0.079	0.020	−4.049	<.001***
Residual length change					
Source		1.342	0.854	1.572	.117
Destination		−0.969	0.947	−1.023	.307
Source × Destination		−0.244	1.275	−0.191	.849
Cage (Nested within destination)	# 2	0.846	0.986	0.858	.392
	# 3	−1.108	0.872	−1.270	.205
	# 4	−0.661	1.105	−0.654	.514
	# 5	−1.010	0.872	−1.158	.248
	# 10	−2.209	0.845	−2.614	.009**
	# 11	−0.208	0.872	−0.239	.811
	# 12	−3.381	0.854	−3.960	<.001***
	# 13	−2.500	0.882	−2.834	.005**
(Intercept)		0.690	0.610	1.131	.259

*Note*: *p* values below .1 are highlighted in bold. *p* values <.05 and ≥.01 are indicated with a single asterisk, <.01 and ≥.001 with two asterisks and <.001 with three asterisks. Rows highlighted in blue represent interaction combinations that address the question of whether the fitness proxy measure in question relates to transplant treatment.

### 3.2 Testing relationships between growth and shape

**FIGURE 4:**
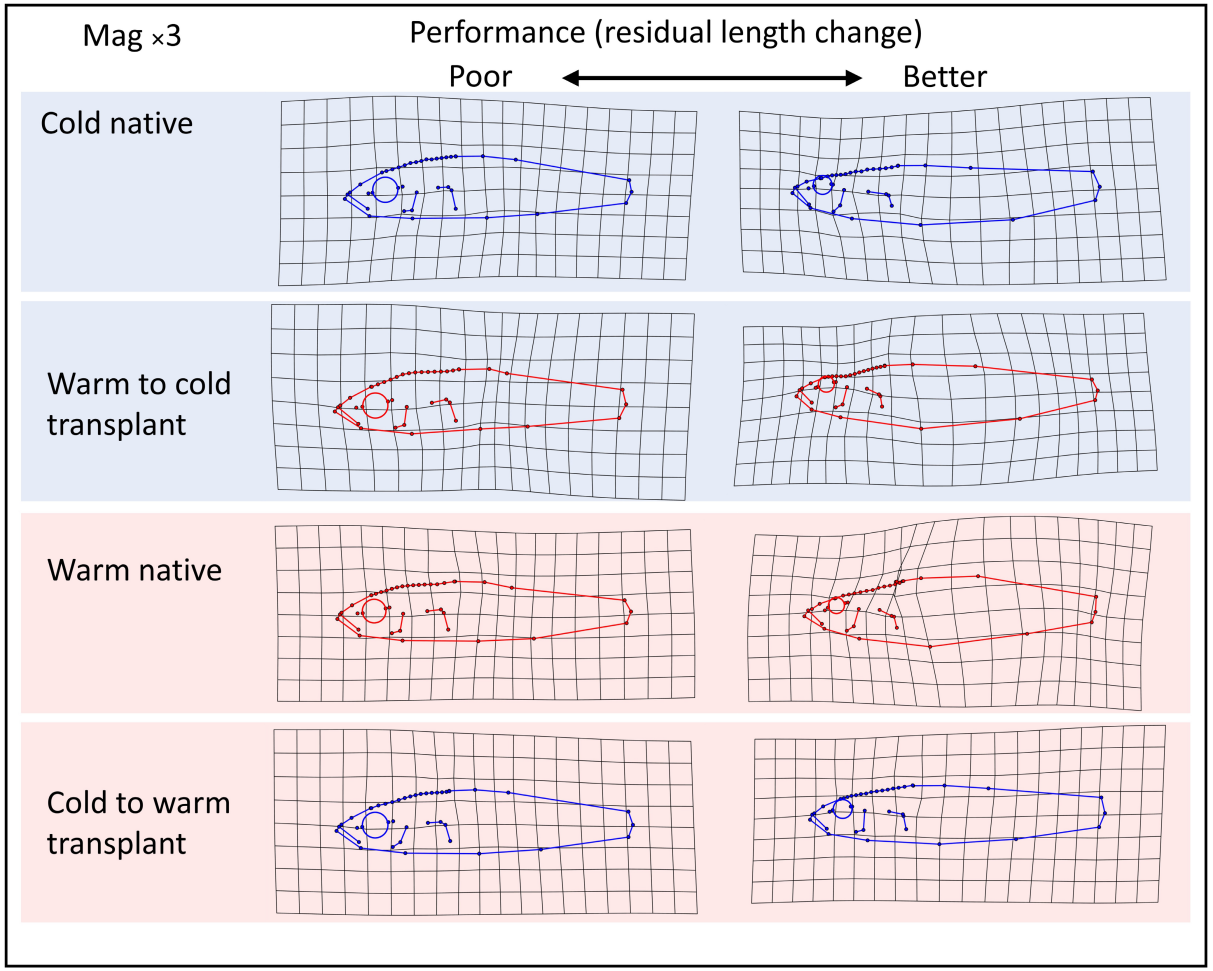
Deformation grids (with 3× magnification) visualising the worst (left) and best (right) performing fish, in terms of residual length change, across the transplant groups. Blue and red outlines represent cold and warm sourced sticklebacks respectively, while blue and red backgrounds represent the cold and warm destination habitats, respectively.

The thermal habitat affected length change (i.e. growth) of warm and cold source fish differently on the basis of head, posterior body and total body shape (indicated by a three‐way interaction between source, destination and the three subdivisions of shape) (Table 3). Fish transplanted to the non‐native habitat appeared to experience greater increases in length if their body shape resembled that of fish native to that habitat (Figure 4). Warm sourced fish with the greatest length increases in the cold habitat were those with narrower bodies, a more upward facing mouth and a concave forehead shape (Figure 4). Cold sourced fish transplanted to the warm habitat showed greater length increases with a slightly more subterminal mouth as compared to cold native fish (Figure 4).

**TABLE 3:** Results of Procrustes ANOVA shape analysis models with growth measures, for total fish, head and body shape of threespine stickleback in Iceland.

Growth measure and variables	Total fish shape						Head shape						Posterior body shape					
	df	*F*	*Z*	SS	PVE	*p*	df	*F*	*Z*	SS	PVE	*p*	df	*F*	*Z*	SS	PVE	*p*
Residual weight change (WC)	1	0.602	−0.621	0.0010	0.2	.732	1	0.307	−1.264	0.0015	0.1	.896	1	0.721	−0.338	0.0009	0.2	.621
Source	1	1.024	0.352	0.0017	0.3	.340	1	0.546	−0.461	0.0026	0.2	.666	1	1.278	0.751	0.0016	0.4	.220
Destination (Dest)	1	0.587	−0.687	0.0010	0.2	.746	1	1.253	0.741	0.0060	0.4	.245	1	0.647	−0.561	0.0008	0.2	.709
Log centroid size (logCS)	1	2.738	2.032	0.0046	0.9	.022*	1	0.791	0.101	0.0038	0.3	.464	1	4.251	3.343	0.0055	1.4	.001**
WC × Source	1	0.529	−0.753	0.0010	0.2	.783	1	0.271	−1.556	0.0013	0.1	.944	1	0.906	0.061	0.0012	0.3	.469
WC × Dest	1	1.360	0.869	0.0023	0.5	.183	1	1.201	0.694	0.0057	0.4	.253	1	0.941	0.099	0.0012	0.3	.462
Source × Dest	1	1.469	1.012	0.0024	0.5	.170	1	2.201	1.394	0.0105	0.8	**.088**	1	0.383	−1.564	0.0005	0.1	.935
WC × logCS	1	0.612	−0.587	0.0010	0.2	.724	1	0.318	−1.208	0.0015	0.1	.886	1	0.713	−0.359	0.0009	0.2	.631
Source × logCS	1	1.017	0.340	0.0017	0.3	.349	1	0.547	−0.461	0.0026	0.2	.667	1	1.250	0.708	0.0016	0.4	.231
Dest × logCS	1	0.612	−0.662	0.0010	0.2	.736	1	1.223	0.714	0.0058	0.4	.253	1	0.631	−0.611	0.0008	0.2	.724
WC × Source × Dest	1	1.017	1.081	0.0025	0.5	.142	1	1.349	0.812	0.0065	0.5	.227	1	1.372	0.850	0.0018	0.4	.206
WC × Source × logCS	1	0.595	−0.724	0.0010	0.2	.776	1	0.284	−1.479	0.0014	0.1	.929	1	0.891	0.031	0.0012	0.3	.480
WC × Dest × logCS	1	1.390	0.895	0.0025	0.5	.174	1	1.23	0.729	0.0059	0.4	.246	1	0.930	0.077	0.0012	0.3	.470
Source × Dest × logCS	1	1.482	1.028	0.0025	0.5	.164	1	2.146	1.368	0.0103	0.8	**.089**	1	0.370	−1.628	0.0005	0.1	.942
WC × Source × Dest × logCS	1	1.548	1.099	0.0026	0.5	.140	1	1.365	0.828	0.0065	0.5	.221	1	1.351	0.819	0.0017	0.4	.215
Residuals	253			0.4200	85.0		253			1.2088	89.9		253			0.3257	82.5	
Residual length change (LC)	1	2.616	1.993	0.0043	0.9	.023*	1	0.793	0.170	0.0038	0.3	.449	1	4.369	3.100	0.0052	1.3	.001**
Source	1	2.119	1.603	0.0035	0.7	.047*	1	0.657	−0.134	0.0031	0.2	.546	1	4.201	3.030	0.0050	1.3	.002**
Destination (Dest)	1	1.883	1.355	0.0031	0.6	**.093**	1	0.339	−1.136	0.0016	0.1	.875	1	4.601	3.506	0.0055	1.4	.001**
Log centroid size (logCS)	1	5.155	3.165	0.0084	1.7	.002**	1	1.128	0.626	0.0054	0.4	.299	1	16.626	6.652	0.0199	5.01	.001**
LC × Source	1	2.164	1.661	0.0035	0.7	**.052**	1	1.045	0.546	0.0050	0.4	.301	1	3.174	2.606	0.0038	1.0	.006**
LC × Dest	1	2.192	1.613	0.0036	0.7	**.063**	1	1.938	1.286	0.0092	0.7	.100	1	3.021	2.425	0.0036	0.9	.007**
Source × Dest	1	1.192	0.581	0.0019	0.4	.297	1	0.446	−0.699	0.0021	0.2	.748	1	2.209	1.684	0.0025	0.6	.048*
LC × logCS	1	2.513	1.922	0.0041	0.8	.028*	1	0.851	0.272	0.0041	0.3	.404	1	4.520	3.116	0.0054	1.4	.001**
Source × logCS	1	2.112	1.596	0.0034	0.7	.048*	1	0.674	−0.097	0.0032	0.2	.528	1	4.145	2.998	0.0050	1.3	.002**
Dest × logCS	1	1.856	1.328	0.0030	0.6	**.097**	1	0.345	−1.111	0.0016	0.1	.869	1	4.522	3.464	0.0054	1.4	.001**
LC × Source × Dest	1	2.394	1.731	0.0039	0.8	.048*	1	2.770	1.669	0.0132	1.0	.044*	1	3.176	2.629	0.0038	1.0	.005**
LC × Source × logCS	1	2.194	1.691	0.0036	0.7	.048*	1	1.130	0.647	0.0054	0.4	.278	1	3.280	2.707	0.0039	1.0	.005**
LC × Dest × logCS	1	2.098	1.536	0.0034	0.7	**.064**	1	1.960	1.303	0.0093	0.7	.101	1	3.026	2.411	0.0036	0.9	.008*
Source × Dest × logCS	1	1.176	0.558	0.0019	0.4	.302	1	0.457	−0.665	0.0022	0.2	.732	1	2.043	1.643	0.0025	0.6	**.053**
LC × Source × Dest × logCS	1	2.207	1.602	0.0036	0.7	**.059**	1	2.730	1.658	0.0130	1.0	.043*	1	2.943	2.457	0.0035	0.9	.008*
Residuals	253			0.4118	83.4		252			1.2035	89.5		253			0.3029	76.8	

*Note*: *p* values below .1 are highlighted in bold. *p* values <.05 and ≥.01 are indicated with a single asterisk, <.01 and ≥.001 with two asterisks and <.001 with three asterisks. Rows highlighted in blue represent interaction combinations that address the question of whether shape relates to growth and transplant treatment.

### 3.3 Testing relationships between growth and allometry

Evidence of divergent shape allometry was detected in the total fish shape and body shape landmark sets (Table 4). For body shape, the clearest difference in allometry between warm and cold sourced fish was seen to be a greater increase in body depth with increasing size in warm sourced (Figure S2a). Allometric divergence was further supported by the finding that size did not differ between warm and cold source fish for both centroid size (*t*_(293)_ = −1.476, *p* = .14) and length (*t*_(293)_ = −1.135, *p* = .26). Warm and cold source fish also did not differ in the amount of variation in centroid size (*F*_(298)_ = 0.560, *p* = .455) or starting length (*F*_(298)_ = 0.950, *p* = .331).

**TABLE 4:** Unique versus common allometry model ANOVA comparison tests for total fish, head and body shape of threespine stickleback in Iceland, using all fish and survivors only data sets.

	Total fish shape					Head shape					Body shape				
	df	*F*	*Z*	SS	*p*	df	*F*	*Z*	SS	*p*	df	*F*	*Z*	SS	*p*
Log centroid size as size															
All fish	1	2.370	1.693	0.0042	**.053**	1	1.103	0.551	0.0056	.302	1	2.512	2.049	0.0033	.022*
Survivors only	1	1.69	1.187	0.0029	.121	1	2.961	1.6246	0.0145	**.051**	1	1.624	1.148	0.0021	.112
Log length as size															
All fish	1	2.574	1.820	0.0045	.04*	1	1.364	0.787	0.0070	.236	1	3.648	2.708	0.0049	.002**
Survivors only	1	0.284	−2.157	0.0005	.984	1	0.429	−0.830	0.0021	.779	1	0.246	−2.297	0.0004	.990

*Note*: *p* values below .1 are highlighted in bold. *p* values <.05 and ≥.01 are indicated with a single asterisk, <.01 and ≥.001 with two asterisks and <.001 with three asterisks. Common allometry model used as null; significant *p* value suggests unique allometry model is most appropriate.

Our data also indicated that survival was related to allometry. Indeed, the detection of divergent allometry was dependant on survival outcome (Table 4). For example, divergent head allometry was detected between warm and cold source *surviving* fish, but not in the *full set* of experimental fish. This may indicate that fish with more intermediate size‐shape relationships died during the experiment, suggesting that our experiment promoted divergent selection on head allometry. Deformation grids depicting allometry indicated that larger surviving warm sourced fish had a more subterminal craniofacial region relative to larger cold sourced fish (Figure S2b). Differences in eye size between small and large fish also appeared to be more distinct in the surviving fish than in those that died. Size‐shape relationships in total fish shape and in body shape show an opposite relationship, where unique allometry was detected in the full set of experimental fish, but not detected when fatalities were removed from the dataset.

Overall, as we reported above the rate of mortality was low during the experiment. This limited follow‐up analysis further tested relationships between allometry and survival in the cold source to warm destination transplant treatment group (lowest survival rate of 74.7%). Allometry was associated with survival in the cold source to warm destination transplant group (Table 5). Here, Procrustes ANOVAs indicated that survival was related to total fish and body shape when interacting with size (for both log centroid size and log length). Among our factors this interaction explained the most variation in the total fish shape model (3.1%–3.3% percentage variation explained (PVE)), and the second most in the body shape model (3.0% PVE) (Table 5). Survivors and fatalities did not differ in centroid size (*t*_(26)_ = 0.255, *p* = .80) or starting length (*t*_(26)_ = 0.312, *p* = .76) indicating that this finding was due to a relationship between size and shape. Also, variation in centroid size did not differ between survivors and fatalities (*F*_(73)_ = 0.839, *p* = .363), nor did starting length (*F*_(73)_ = 1.017, *p* = .316) indicating this finding was not driven by a lack of variation in one group.

**TABLE 5:** Results of Procrustes ANOVA shape analysis of the cold to warm transplant group, with survival and log centroid size as explanatory variables.

	Total fish shape						Head shape						Body shape					
	df	*F*	*Z*	SS	PVE	*p*	df	*F*	*Z*	SS	PVE	*p*	df	*F*	*Z*	SS	PVE	*p*
Log centroid size as size																		
Log CS	1	1.312	0.752	0.0028	1.7	.244	1	3.539	1.844	0.0249	4.7	.027*	1	1.116	0.418	0.0011	1.4	.331
Survival	1	2.331	1.642	0.0051	3.1	**.058**	1	2.376	1.434	0.0167	3.1	**.088**	1	2.504	2.021	0.0024	3.2	.021*
Log CS × Survival	1	2.332	1.641	0.0051	3.1	**.056**	1	2.335	1.415	0.0164	3.1	**.089**	1	2.541	2.053	0.0025	3.3	.021*
Residuals	71			0.1454	94.2		71			0.5000	93.5		71			0.0692	91.4	
LOG length as size																		
Log *L*	1	1.323	0.770	0.0029	1.8	.236	1	1.925	1.184	0.0139	2.6	.140	1	1.259	0.667	0.0013	1.7	.256
Survival	1	2.424	1.710	0.0053	3.2	.048*	1	1.567	0.952	0.0113	2.1	.198	1	2.292	1.815	0.0023	3.0	.033*
Log *L* × Survival	1	2.435	1.704	0.0053	3.2	.048*	1	1.541	0.934	0.0111	2.1	.203	1	2.328	1.848	0.0023	3.1	.028*
Residuals	71			0.1545	94.4		71			0.5118	95.7		71			0.0708	93.4	

*Note*: *p* values below .1 are highlighted in bold. *p* values <.05 and ≥.01 are indicated with a single asterisk, <.01 and ≥.001 with two asterisks and <.001 with three asterisks. Rows highlighted in blue represent interaction combinations that address the question of whether shape relates to survival and log centroid size.

**FIGURE 5:**
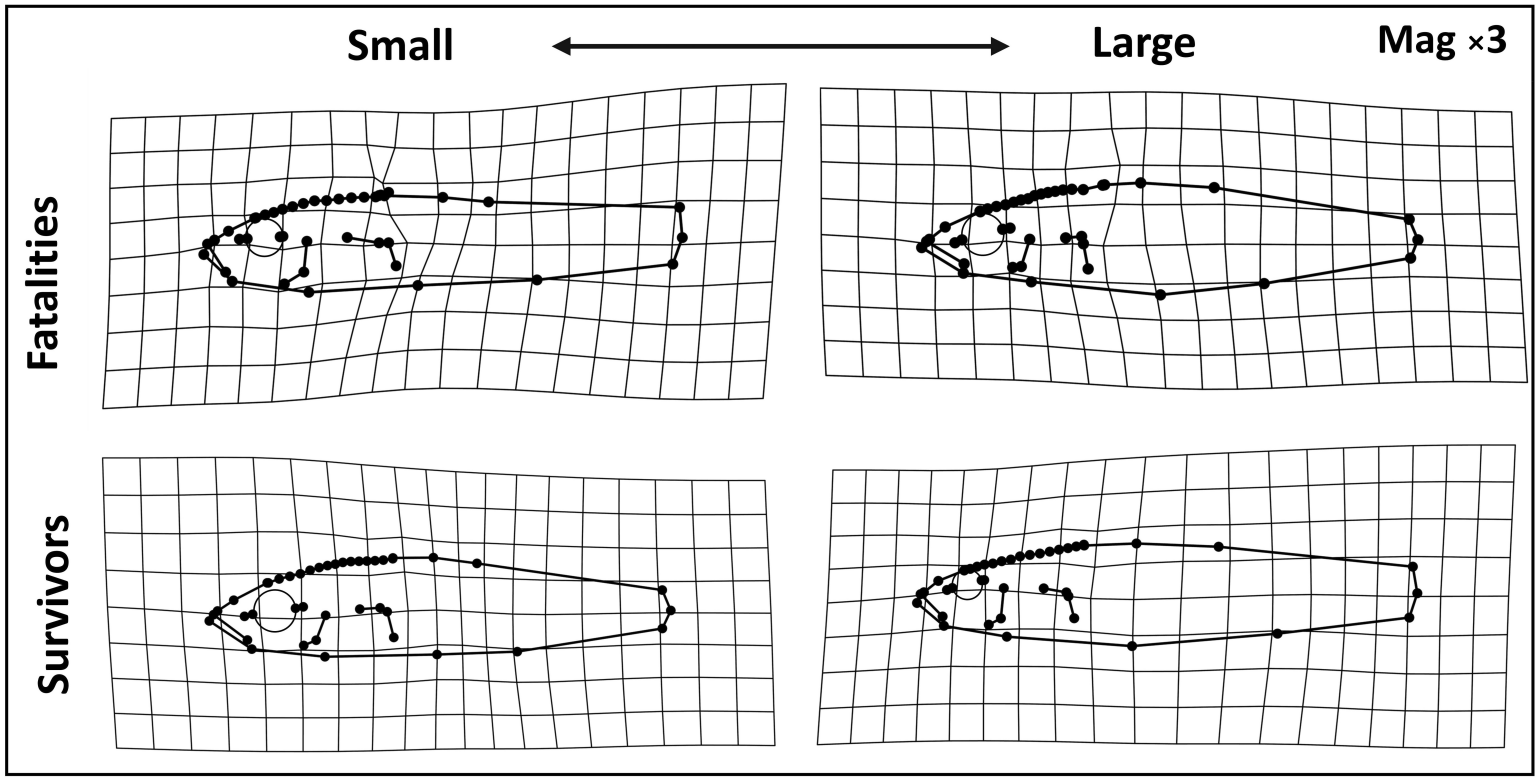
Deformation grids (with 3× magnification) depicting allometric relationships as shape extremes related to centroid size for the surviving and non‐surviving cold source to warm habitat transplant fish.

Larger cold to warm survivors possessed a greater body depth, shorter craniofacial region and a smaller eye than larger fatalities, while small survivors had a more fusiform body profile and larger eye than smaller fatalities (Figure 5).

## 4 DISCUSSION

We tested for fitness proxy consequences of divergence between warm and cold sourced stickleback and for an effect of divergent morphology on growth in a field reciprocal transplant experiment. We found evidence of negative consequences for fish transplanted to the non‐native habitat, with further evidence suggesting that divergent shape and allometry play a role in how well fish perform.

### 4.1 Testing for evidence of adaptive divergence

Our findings support our first prediction that stickleback should perform best in their native habitat, suggesting that adaptive divergence has occurred (Blanquart et al., 2013; De Villemereuil et al., 2016). However, there was evidence for an asymmetric effect of thermal habitat adaptation as cold fish transplanted to the warm habitat had reduced survival and increased parasite prevalence, while warm fish transplanted to the cold habitat did not, only suffering an increased chance of weight loss. Similar results in the costs of migration have been found for river and lake stickleback, where fish transplanted to either habitat suffered fitness consequences, but in different ways (Kaufmann et al., 2017).

The poorer outcomes for cold sourced stickleback transplanted to the warm habitat may be due, in part, to immunological challenges. The higher temperatures of the warm habitat better match the thermal optima of *S. solidus*, encouraging its growth and potentially dampening the host's immune system functioning (Franke et al., 2019). Indeed, transplant treatment was found to affect *S. solidus* prevalence with a higher infection rate found in both warm and cold sourced fish in the warm habitat. Parasite infection was found to increase weight gain, likely due to the growth of the parasite rather than that of the fish. While it was not possible to establish infection status prior to transplantation, the warm habitat may induce greater infection by parasites (possibly there may be a greater abundance of the first‐intermediate host (cyclopoida copepods), or an induced change in stickleback foraging strategy that increases consumption of copepods) or facilitate faster parasite growth, either of which is likely to have a fitness consequence (Barber et al., 2004; Franke et al., 2019; Grécias et al., 2018; Heins & Baker, 2003). Such differences in parasite–host dynamics between habitats could reinforce adaptive divergence and local adaptation (Kaufmann et al., 2017). We also found that cold sourced fish transplanted to the warm habitat were more likely to be infected at the end of the experiment than warm native fish. It is unlikely that this difference was simply due to pre‐experiment infection status as all fish within the cold habitat were less likely to be infected than those in the warm habitat. Thus, cold sourced fish in the warm habitat are possibly more susceptible to infection, or less able to suppress parasite growth than the warm native fish. This may be due to differences in immunological systems between warm and cold sourced stickleback, or an additional effect caused by the stress of the warm habitat. In either case, this result indicates a fitness consequence for cold sourced fish transplanted to the warm habitat and a sign that warm sourced sticklebacks have adapted to their native conditions. However, not all of our findings can be attributed to parasites as even after accounting for parasite effects, warm sourced fish in their non‐native (cold) habitat are more likely to lose weight than cold sourced fish in their non‐native (warm) habitat (Figure 3d). This may indicate differences in the quality of the thermal habitats or potentially locally adapted metabolic differences between fish from different source habitats (Pilakouta et al., 2020).

Asymmetric effects may also arise from the different seasonal temperature variations experienced by the two populations (see Table S1). The warm and cold habitats differ in temperature by around 10°C consistently throughout the year; as a result, the winter temperature of the warm habitat is close to the summer temperature of the cold habitat. Therefore, while cold sourced stickleback transplanted to the warm habitat (in a summer experiment) are experiencing a novel temperature, warm sourced fish transplanted to the cold habitat experience temperatures close to what they experience in winter. This suggests that the degree of ‘novelty’ experienced by the two source populations would differ in our experiment. Indeed, *variation* in weight change (as shown by the spread of the boxplots in Figure 3a) was much greater in cold fish in the warm habitat, potentially suggesting that these fish were experiencing a more novel habitat.

Alternatively, asymmetric effects may be caused by evolutionary history. As the warm habitat was likely colonised by stickleback originating from the cold habitat, the transplantation of warm sourced stickleback to the cold habitat represented a return to the ancestral environment. Similarly, reciprocal transplants using chicken (*Gallus gallus domesticus*) populations adapted to different altitudes found asymmetric costs of transplantation, with high‐altitude chickens showing little reduction in fitness when transplanted to the ancestral low‐altitude habitat (Ho et al., 2020). Gene expression also showed that high‐altitude chickens were able to adjust through plasticity to match the native low‐altitude profile more closely, while low‐altitude chickens in the high‐altitude habitat could not. Thus, phenotypic plasticity may play a role in rapid re‐adaptation to ancestral environments by enabling organisms to adapt to a habitat experienced by their ancestors more easily (Parsons et al., 2020; Rajakumar et al., 2012).

### 4.2 Testing relationships between growth and shape

We found evidence supporting prediction 2, that divergent shape between thermal habitats would relate to growth, and prediction 3, that transplanted fish will grow best in the alternate habitat when they possess a body shape similar to native fish. Several aspects of shape from the best‐performing fish in each transplant group were similar to the typical native shape for each habitat as described by Pilakouta et al. (2023), where warm sourced stickleback were found to have deeper bodies, a more subterminal mouth and steeper craniofacial profiles. In our experiment, the cold habitat appeared to favour a narrower body depth and a concave profile along the neurocranium (Figure 4). This morphology suggested reduced jaw musculature and potentially different foraging and swimming modes relative to the optimum shape in the warm habitat (McGee et al., 2013). The warm habitat appeared to favour a more subterminal mouth, in line with a shift to a more benthic lifestyle (Figure 4) (Willacker et al., 2010). These results suggest that the heritable differences in shape between warm and cold stickleback are adaptive in nature. However, while shape was related to growth, the effect sizes were very small (Table 3). We note that this was a short‐term experiment, and we would not necessarily expect large effects to accrue over a month. Indeed, previous research suggests that even small changes in phenotypic variation can have large fitness effects over a long period. For example, bill shape polymorphism in the African estrildid finch (*Pyrenestes ostrinus*) shows that a difference of less than 1 mm in bill length can alter fitness by more than 50% (Smith, 1990). For our experiment we could expect a cumulative effect over a longer period of time, especially during the reproductive season or winter when food availability is low. Further investigation over a different or longer period of time may therefore reveal more about the potential for stickleback to adapt to thermal habitats.

A more benthic morphology in warm habitats could be driven by a shift away from limnetic prey, possibly reflecting differences in prey availability or a dietary change to prioritise higher value prey. Invertebrate communities have been found to differ between geothermal and cold habitats, with geothermal habitats typically being dominated by large, benthic macroinvertebrates such as gastropods and chironomid larvae (Nelson et al., 2017; O'Gorman et al., 2012; Scrine et al., 2017). Additionally, the warm habitat is shallower than the cold habitat and so the space for a limnetic habitat is reduced. Limnetic prey may therefore be less available to geothermal stickleback, inducing a shift in diet towards benthic prey. This potential dietary shift could also be reflective of the selective pressures of the warm habitat, with higher temperatures increasing metabolic rate, and so increasing nutritional requirements and the risk of starvation (Fry, 1967). Indeed, dietary differences and shifts in feeding strategy occur in geothermal populations of brown trout (*Salmo trutta*) that prefer prey that is higher in the trophic web even when such prey items may be relatively rare (O'Gorman et al., 2016). Also, previous research from Áshildarholtsvatn shows that when temperature is increased warm source fish experience a smaller increase in metabolic rate relative to their cold source counterparts (Pilakouta et al., 2020). Therefore, cold migrants to the warm habitat should experience an elevated metabolic rate higher than the native fish increasing the risk of starvation. Dietary shifts between thermal habitats may also impact *S. solidus* prevalence, as stickleback become infected when they eat an infected copepod, which is typically limnetic. Dietary shifts away from limnetic prey, whether due to abundance or metabolic needs, could therefore disrupt the route of transmission of the parasite to the stickleback. However, as shown here, *S. solidus* is particularly prevalent in fish housed in the warm habitat, suggesting that geothermal stickleback are indeed consuming infected copepods and so at least part of their diet is made up of limnetic prey.

### 4.3 Testing relationships between growth and allometry

Support for adaptive allometric divergence was found for our fourth prediction. Divergent allometry between warm and cold populations was related to growth, suggesting an adaptive role (Table 4 and Figure S2); however, the effect size of this relationship was very small (Table 4). This small effect size may have a larger impact in an experiment performed over a longer period of time, or with the inclusion of harsher seasons. Though it is possible that some body shape variation could be attributed to the growth of *S. solidus* distending the abdomen, this would not explain how head shape allometry (a trait which would not be affected by *S. solidus*) interacts with length change (Table 3). Diverging allometry in a system which is no older than 70 years may be a surprising result, but some allometric relationships can be quick to evolve (Adams & Nistri, 2010; Bolstad et al., 2015; Frankino et al., 2019; Voje et al., 2013; Voje & Hansen, 2013). It is currently unknown whether this divergent allometry is due to an adaptive plastic response to thermal habitat, or a genetic divergence between warm and cold sourced stickleback. Divergent allometries could partially be driven by plastic temperature effects on growth, with changes in the timing and extent of developmental events being broadly influenced. Thus, while the allometric relationships we have revealed could be due to plasticity, future work examining their heritability will be especially important for determining their evolutionary consequences.

### 4.4 Study limitations

While a field experiment can be powerful for testing adaptive divergence in a natural setting this approach poses some limitations. For example, the density of free‐living stickleback was unknown; thus, the experimental density we used may have impacted our results. Indeed, it is possible that fish density differs between warm and cold habitats, making one type better adapted to higher levels of competition. For this experiment we selected equal densities of 25 sticklebacks per cubic metre across all cages. This value was selected as it is within the range of previous reciprocal transplant experiments involving this species, which ranges from approximately 0.05 sticklebacks per m^3^ used by Räsänen and Hendry (2014) to approximately 133 sticklebacks per m^3^ used by Kaufmann et al. (2017). Additionally, the fish used in this experiment were not yet full sized and so a higher density was possible.

Due to the shallow nature of the warm habitat, cage dimensions were selected for equal volumes between habitats; however, this results in more water surface area available to fish in warm habitat cages which may impact their ability to forage benthically compared to fish housed in cold habitat cages. Such a challenge is common in reciprocal transplant experiments, and differing dimensions of enclosures are often used (Hendry et al., 2002; Räsänen & Hendry, 2014; Stutz et al., 2015). Additionally, the differences in depth between habitats may result in differences in vegetation, and therefore habitat complexity, between the two habitats (Thomaz & da Cunha, 2010). Habitat complexity can interact with morphological variation in fish (Freudiger et al., 2021). An additional limitation of this experiment was the lack of sex information. Sex is known to affect body shape in sticklebacks (Leinonen et al., 2011) and so may play a role in morphological divergence with thermal habitat.

Predation was also prevented but could be relevant for a fish migrating to a different thermal habitat. We also expect that predation would differ between thermal habitats, as piscivorous birds may prefer hunting in warm water (Esler, 1992; Stocking et al., 2018), while salmonid predators would likely avoid them (Santiago et al., 2016). Indeed, Arctic terns (*Sterna paradisaea*) were frequently observed catching fish in the warm habitat, which is shallower than the cold habitat. Also, while each cage was provided with mud and native plants, exactly matching the natural variation between wild habitats would be difficult to achieve in an artificial cage, particularly for the cold habitat, which was larger and deeper and potentially more variable. The complexity of a habitat can affect the optimal shape, for example, a more densely planted and complicated habitat could benefit a deeper body more adept at manoeuvring (Domenici et al., 2008). Finally, this geothermal system differs from climate change–driven temperature increases in that temperature change would have occurred rapidly rather than gradually. Gradual environmental change can result in delayed evolution, due to weaker selective pressure and the potential for phenotypes selected for partway through the process of environmental change to prove to be dead ends, not useful at more extreme degrees of environmental change (Gorter et al., 2015; Guzella et al., 2018). However, adaptation to different rates of environmental change can still result in similar fitness endpoints (Gorter et al., 2015).

## 5 CONCLUSIONS

Our findings suggest that stickleback found in a geothermally warmed system have adapted to their habitat. Therefore, while increasing temperatures will pose challenges, it may be that species with a tendency for phenotypic change could be more persistent under climate change. Warm sourced stickleback suggest a shift to a benthic lifestyle through morphology, with contributions from allometric variation, but also a potential change in immunity. This is notable given that the warm habitat has been established for no more than 70 years. The underlying mechanisms for such changes are likely to be insightful for other populations as a number of traits in fish are known to be plastic in response to temperature (Campbell et al., 2021; Crozier & Hutchings, 2014; Sfakianakis et al., 2011), but it is also the case that adaptive evolutionary divergence in fish can be extremely rapid (Barrett et al., 2011; Kovach et al., 2012) with evidence of heritable divergence in this system (Pilakouta et al., 2023) and some degree of genetic divergence (I. Fisk, G. Lawson‐Duck and K. J. Parsons, Unpublished data). We suspect that a wide range of factors contribute towards adaptive divergence between thermal habitats, an important challenge to discern in a warming world.

## AUTHOR CONTRIBUTIONS

**Bethany A. Smith:** Conceptualization (equal); data curation (lead); formal analysis (lead); investigation (lead); methodology (equal); visualization (lead); writing – original draft (lead); writing – review and editing (equal). **Ana P. B. Costa:** Supervision (supporting); writing – review and editing (supporting). **Bjarni K. Kristjánsson:** Conceptualization (supporting); investigation (supporting); resources (equal); supervision (supporting); writing – review and editing (supporting). **Kevin J. Parsons:** Conceptualization (equal); funding acquisition (lead); investigation (equal); methodology (equal); project administration (lead); resources (lead); supervision (lead); writing – review and editing (lead).

## CONFLICT OF INTEREST STATEMENT

The authors declare no conflicts of interest.

## Supporting information


Appendix S1:

## Data Availability

All data that support the findings of this study is available at the Dryad Digital Repository (https://datadryad.org/stash/share/Bi_naH5RChL48bVhoLnOAb6MSOsUh7fc6tAdpTSHN7U).
