# Supplementary material for: Experimental evidence for adaptive divergence in response to a warmed habitat reveals roles for morphology, allometry and parasite resistance
Source: Ecol Evol. 2024 Feb 7;14(2):e10907. doi: 10.1002/ece3.10907 (PMC10850817; doi:10.1002/ece3.10907)
Supplement: Supplementary file 1 — Appendix S1: [file ECE3-14-e10907-s001.docx]

# Appendix 1: Supplementary materials

Supplementary Table 1 Water chemistry and seasonal temperatures of the warm and cold thermal habitats at Áshildarholtsvatn (measured 2016/17) and temperatures measured during the experiment (21/06/2019 – 18/07/2019)

|  |  |  |  |  |  |  |  |  |  |  |  |  | Temp during experiment | | |
| --- | --- | --- | --- | --- | --- | --- | --- | --- | --- | --- | --- | --- | --- | --- | --- |
| Site | **Coordinates** | **Summer temp**  **(^o^C)** | **Winter temp**  **(^o^C)** | **Dissolved**  **Oxygen (mg/L)** | **TDS (ppm)** | **Sulfide (mg/L)** | **Phosphate (mg/L)** | **Magnesium (mg/L)** | **Iron (mg/L)** | **Silica (mg/L)** | **Hardness Ca2+ (mg/L)** | **Hardness (Trans %)** | **Mean (stdev) (^o^C)** | **Max (^o^C)** | **Min (^o^C)** |
| ASHN warm | 65.72516 -, 19.600725 | 24.1 | 12.5 | 13.4 | 128 | <0.1 | 0.1 | <0.5 | 0.345 | 50.5 | <3.0 | 92.0 | 21.76 (3.09) | 27.5 | 14.0 |
| ASHN cold | 65.724973 -, 19.602784 | 12.2 | 3.4 | 12.2 | 97 | <0.1 | 0.02 | >10.0 | 0.118 | 2.7 | 22.8 | 60.7 | 14.79 (2.17) | 18.7 | 10.7 |

*Supplementary Table 2: Model design iterations used for AIC model selection. For each analysis the model with the lowest AIC value was selected, unless a simpler model had an AIC within 2 of the lowest AIC model. Final selected models are highlighted in blue and bold.*

| Survival analysis | | | |
| --- | --- | --- | --- |
| *Model name* | *df* | *AIC* | *Model design* |
| Model 1 | **4** | **182.07** | **Survival ~ source*destination** |
| Model 2 | 12 | 193.27 | Survival ~ source*destination + cage (nested within destination) |
| Model 3 | 8 | 181.26 | Survival ~ source*destination*starting weight |
| Model 4 | 5 | 183.71 | Survival ~ source*destination + starting weight |
| Model 5 | 8 | 181.16 | Survival ~ source*destination*starting length |
| Model 6 | 5 | 182.90 | Survival ~ source*destination + starting length |
| Model 7 | 6 | 184.26 | Survival ~ source*destination + starting length + starting weight |
| Model 8 | 7 | 185.60 | Survival ~ source*destination + starting length*starting weight |
| Model 9 | 16 | 193.48 | Survival ~ source*destination*starting weight + cage (nested within destination) |
| Model 10 | 13 | 195.27 | Survival ~ source*destination + starting weight + cage (nested within destination) |
| Model 11 | 16 | 193.75 | Survival ~ source*destination*starting length + cage (nested within destination) |
| Model 12 | 13 | 195.99 | Survival ~ source*destination + starting length + cage (nested within destination) |
| Model 13 | 14 | 196.22 | Survival ~ source*destination + starting length + starting weight + cage (nested within destination) |
| Model 14 | 15 | 197.54 | Survival ~ source*destination + starting length*starting weight + cage (nested within destination) |
| Model 15 | 25 | 215.29 | Survival ~ source*destination*starting length*starting weight + cage (nested within destination) |
|  |  |  |  |
| Parasite analysis | | | |
| *Model name* | *df* | *AIC* | *Model design* |
| Model 1 | 4 | 288.04 | Parasite infection status ~ source*destination |
| Model 2 | 12 | 299.60 | Parasite infection status ~ source*destination +cage (nested within destination) |
| Model 3 | 8 | 292.96 | Parasite infection status ~ source*destination*starting weight |
| Model 4 | 5 | 287.88 | Parasite infection status ~ source*destination + starting weight |
| Model 5 | 8 | 289.07 | Parasite infection status ~ source*destination*starting length |
| Model 6 | 5 | 286.41 | Parasite infection status ~ source*destination + starting length |
| Model 7 | 16 | 305.40 | Parasite infection status ~ source*destination*starting weight +cage (nested within destination) |
| Model 8 | 13 | 300.03 | Parasite infection status ~ source*destination + starting weight +cage (nested within destination) |
| Model 9 | 13 | 297.19 | Parasite infection status ~ source*destination + starting length +cage (nested within destination) |
| Model 10 | 16 | 299.94 | Parasite infection status ~ source*destination*starting length +cage (nested within destination) |
| Model 11 | 16 | 297.62 | Parasite infection status ~ source*destination*starting weight*starting length |
| Model 12 | **7** | **282.40** | **Parasite infection status ~ source*destination + starting weight*starting length** |
| Model 13 | 15 | 294.62 | Parasite infection status ~ source*destination + starting weight*starting length +cage (nested within destination) |
| Model 14 | 24 | 309.95 | Parasite infection status ~ source*destination*starting weight*starting length +cage (nested within destination) |
| Model 15 | 14 | 297.66 | Parasite infection status ~ source*destination + starting weight + starting length +cage (nested within destination) |
|  |  |  |  |
| Weight change analysis | | | |
| *Model name* | ***df*** | ***AIC*** | ***Model design*** |
| Model 1 | 5 | -444.44 | Residual weight change ~ source*destination |
| Model 2 | 13 | -462.78 | Residual weight change ~ source*destination + cage (nested within destination) |
| Model 3 | 17 | -480.42 | Residual weight change ~ source*destination*Parasite infection status + cage (nested within destination) |
| Model 4 | **14** | **-486.42** | **Residual weight change ~ source*destination + cage (nested within destination) + Parasite infection status** |
| Model 5 | 9 | -460.01 | Residual weight change ~ source*destination*Parasite infection status |
| Model 6 | 6 | -465.85 | Residual weight change ~ source*destination + Parasite infection status |
|  |  |  |  |
| Length change analysis | | | |
| *Model name* | ***df*** | ***AIC*** | ***Model design*** |
| Model 1 | 5 | 1379.12 | Residual length change ~ source*destination |
| Model 2 | **13** | **1364.98** | **Residual length change ~ source*destination + cage (nested within destination)** |
| Model 3 | 14 | 1366.88 | Residual length change ~ source*destination + cage (nested within destination) + Parasite infection status |
| Model 4 | 6 | 1380.87 | Residual length change ~ source*destination + Parasite infection status |
| Model 5 | 9 | 1385.21 | Residual length change ~ source*destination*Parasite infection status |
| Model 6 | 17 | 1371.77 | Residual length change ~ source*destination*Parasite infection status + cage (nested within destination) |
|  |  |  |  |


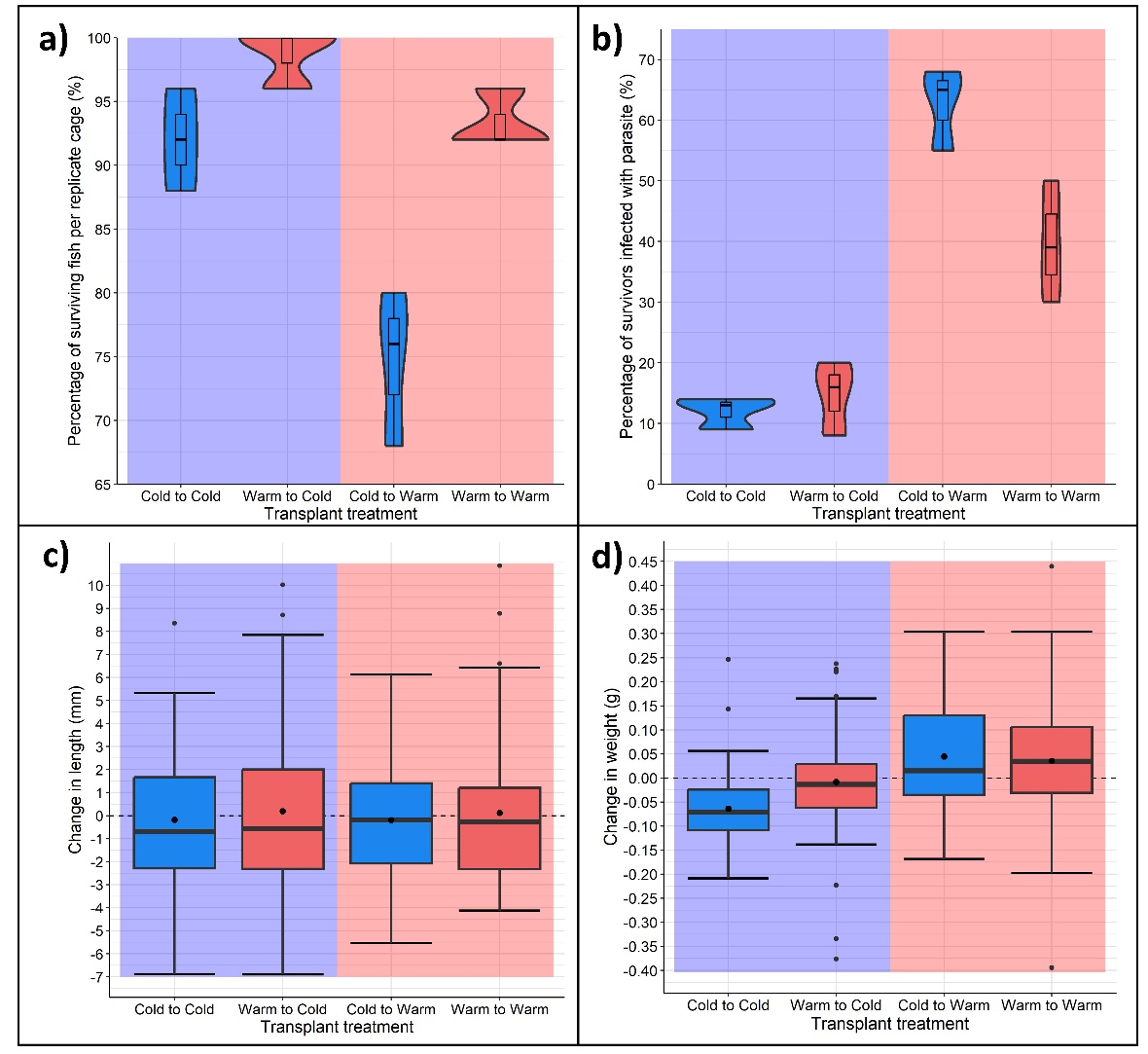


Supplementary Figure 1. Violin and box and whisker plots displaying variation in a) the mean percentage of surviving fish per cage per treatment, b) the percentage of surviving fish found to be infected with S. solidus, c) the residual change in length and d) residual weight changes (g) adjusted for parasite and cage effects, that occurred with experimental treatments in the reciprocal transplant experiment involving natural cold and warm habitats. Top and bottom hinges represent 25^th^ and 75^th^ percentile, centreline represents 50^th^. Black dot displays mean residual weight change. Whiskers give 95% confidence interval. Blue and red filled boxes represent the cold and warm sourced fish respectively, blue and red backgrounds represent the cold and warm destination habitats, respectively.


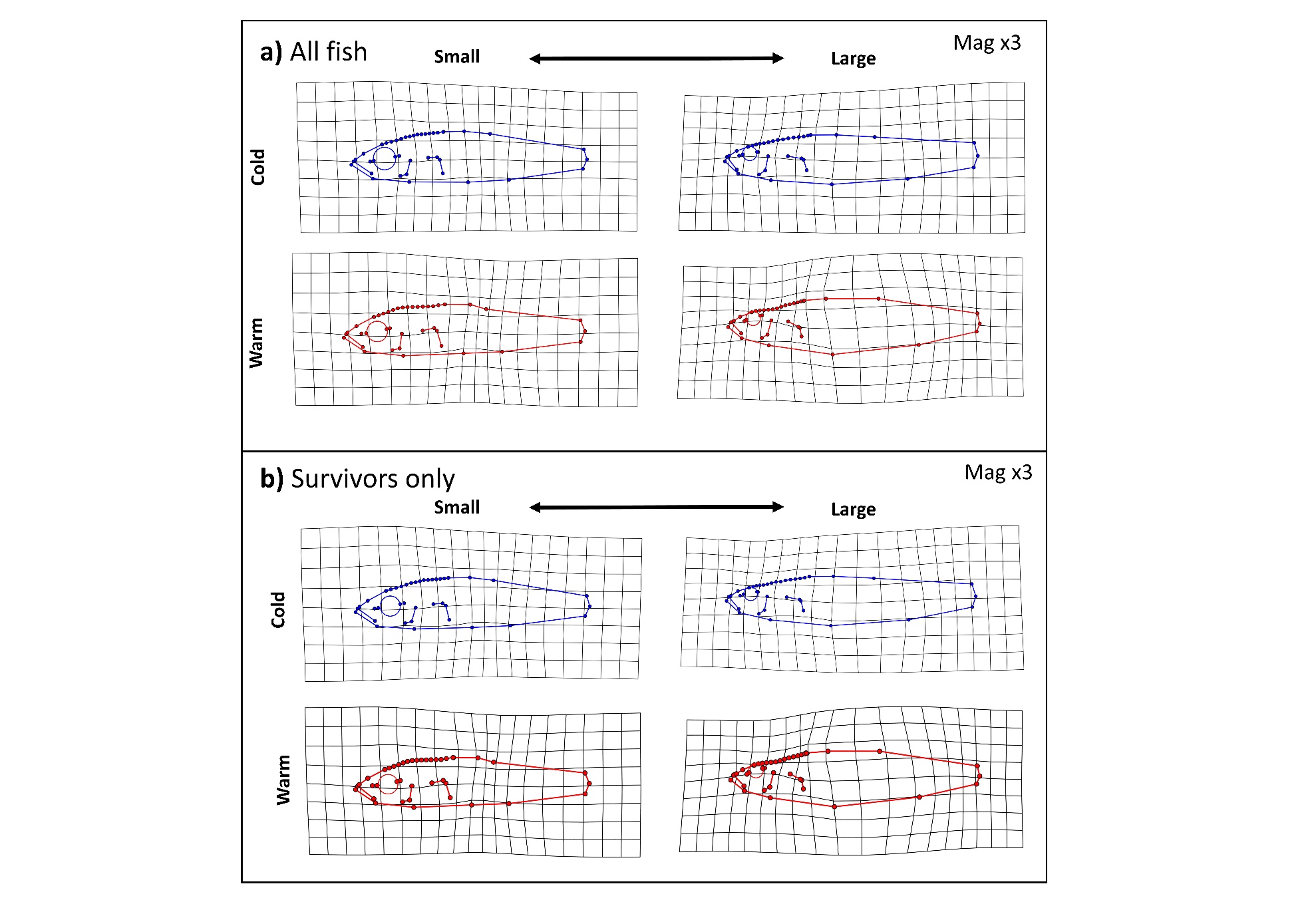


Supplementary figure 2. Deformation grids (with 3x magnification) depicting the allometric shape variation (with centroid size as size) of warm and cold source fish for datasets of a) all fish used in this experiment and b) surviving fish only. Here relationship between centroid size and shape are plotted to the extremes of size.
